# Inhibition of Cellular Protein Secretion by Norwalk Virus Nonstructural Protein p22 Requires a Mimic of an Endoplasmic Reticulum Export Signal

**DOI:** 10.1371/journal.pone.0013130

**Published:** 2010-10-18

**Authors:** Tyler M. Sharp, Susana Guix, Kazuhiko Katayama, Sue E. Crawford, Mary K. Estes

**Affiliations:** 1 Department of Molecular Virology and Microbiology, Baylor College of Medicine, Houston, Texas, United States of America; 2 Virology II, National Institute of Infectious Diseases, Tokyo, Japan; University of Texas Medical Branch, United States of America

## Abstract

Protein trafficking between the endoplasmic reticulum (ER) and Golgi apparatus is central to cellular homeostasis. ER export signals are utilized by a subset of proteins to rapidly exit the ER by direct uptake into COPII vesicles for transport to the Golgi. Norwalk virus nonstructural protein p22 contains a YXΦESDG motif that mimics a di-acidic ER export signal in both sequence and function. However, unlike normal ER export signals, the ER export signal mimic of p22 is necessary for apparent inhibition of normal COPII vesicle trafficking, which leads to Golgi disassembly and antagonism of Golgi-dependent cellular protein secretion. This is the first reported function for p22. Disassembly of the Golgi apparatus was also observed in cells replicating Norwalk virus, which may contribute to pathogenesis by interfering with cellular processes that are dependent on an intact secretory pathway. These results indicate that the ER export signal mimic is critical to the antagonistic function of p22, shown herein to be a novel antagonist of ER/Golgi trafficking. This unique and well-conserved human norovirus motif is therefore an appealing target for antiviral drug development.

## Introduction

Maintenance of cellular homeostasis is directly dependent on the proper functioning of the Golgi apparatus, which is central to lipid trafficking and protein secretion. Protein trafficking from the endoplasmic reticulum (ER) to the Golgi is mediated by vesicles coated in COPII protein complexes, whereas the retrograde Golgi-to-ER pathway is mediated by COPI-coated vesicles [1]. Upon export from the ER at ER exit sites (ERES), cellular proteins accumulate and traffic into budding COPII vesicles, which are minimally composed of the GTPase Sar1 and heteromeric complexes of Sec13/31 and Sec23/24 [2], [3]. COPII vesicles then traffic along microtubules through the ER/Golgi intermediate compartment to the *cis* Golgi, where vesicles lose their COPII coat, fuse with the Golgi, and progress to the *trans* Golgi [4]–[6]. A subset of cellular and viral proteins that rapidly exit the ER employ either di-hydrophobic [7], di-basic [8] or di-acidic [9], [10] ER export signals that mediate their specific uptake into COPII vesicles by direct interaction with either Sec24 or Sar1 at ERES. Export of proteins from the ER and subsequent trafficking of COPII vesicles to the Golgi is mediated by a number of cellular factors, and proteins of both cellular and microbial origin are known to antagonize this pathway.

Perhaps the most well-known ER/Golgi trafficking antagonist, the fungal metabolite brefeldin A (BFA) targets the GTPase ADP-ribosylation factor 1 (Arf1) responsible for COPI vesicle budding at the Golgi by stabilizing an Arf/Sec7 intermediate during nucleotide exchange [11]. This prevents nucleotide dissociation and ultimately deactivates Arf1 to induce a global inhibition of cellular protein secretion. The 3A proteins encoded by the picornaviruses coxsackievirus B3 (CVB3) and poliovirus (PV) also target Arf1. 3A inhibits GBF1, a guanine exchange factor necessary for Arf1 activition [12], [13], resulting in Golgi disruption and inhibition of protein secretion. Consequently, surface expression of MHC Class I decreases and the normal cytokine release that aids in clearance of infected cells is inhibited [14]–[16]. This results in a prolonged period of viral replication before the infected cell can be cleared by the immune system [12], [15].

Human noroviruses are the causative agent of approximately 23 million annual cases of gastroenteritis in the U.S. and are classified as Category B biodefense pathogens [17], [18]. Noroviruses are composed of five genogroups within the family *Caliciviridae*, and viruses in genogroups I (GI) and II (GII) are the most frequently detected in humans [19]–[21]. Noroviruses code for six nonstructural and two structural proteins [22]; however, one of these proteins, the nonstructural protein p22, has no identified function in any calicivirus, although an early study on the immune response following infection with Norwalk virus (NV), the prototype human norovirus and calicivirus, demonstrated an immune response directed against p22 in convalescent sera [23]. The study of p22 and other human norovirus proteins is complicated by the lack of both an efficient tissue culture system to grow noroviruses and a reverse genetics system to directly examine protein function during viral infection.

Replication of two cultivatable animal caliciviruses, feline calicivirus (FCV) and murine norovirus (MNV), induces cellular membrane rearrangements as well as alterations in Golgi architecture [24], [25], suggesting that Golgi disassembly may be a common consequence of infection. In support of this, FCV p30, a homologue of NV p22, is membrane associated and independently induces ultrastructural changes in several secretory pathway organelles [26], thus proposing ER-derived membranes as a source of membranes to anchor viral genome replication. Similarly, Fernandez-Vega and colleagues demonstrated that the NV nonstructural protein p48 induces Golgi disassembly [27]; however, the possibility of additional viral proteins contributing to alterations in Golgi phenotype and antagonism of protein secretion, as is the case for several picornaviruses [28], [29], has not been examined.

In the current study, we asked if the Golgi rearrangements observed during animal calicivirus and picornavirus replication also occur during human norovirus replication, and if p22 has a role in this process. We discovered that p22 has a highly conserved motif that mimics a traditional di-acidic ER export signal and is required for inhibition of ER/Golgi trafficking. This represents a novel approach to antagonize ER/Golgi trafficking, as no other cellular or microbial protein has been described to use a motif similar to an ER export signal to gain access to and antagonize the secretory pathway.

## Results

### The Golgi apparatus is disassembled during NV replication

**Figure 1:**
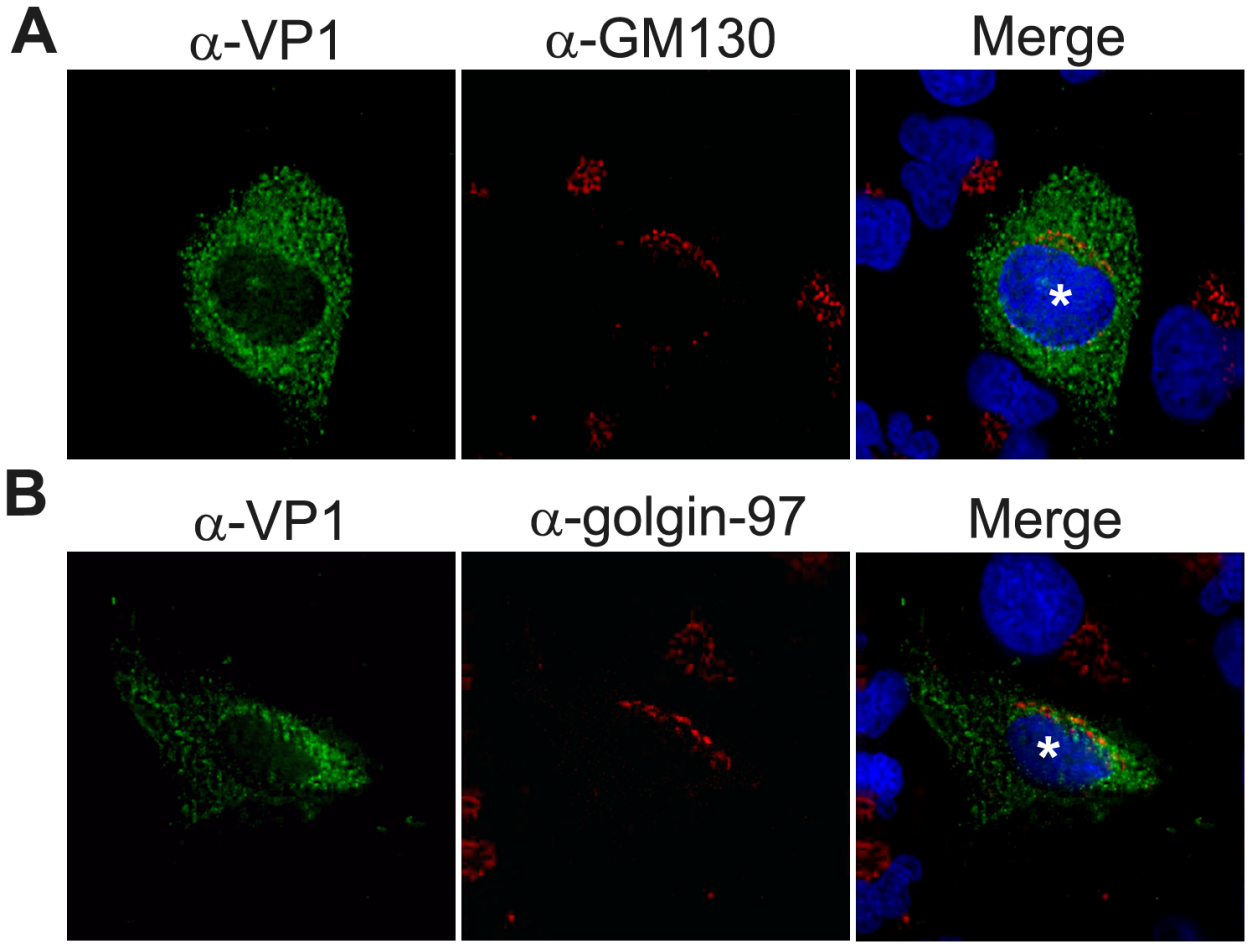
NV replication induces Golgi fragmentation. Viral RNA, purified from the stool of a human volunteer infected with Norwalk virus, was transfected into Huh7 cells grown on coverslips. At 24 hours post-transfection (hpt), cells were fixed and stained for the viral protein VP1 (Alexa 488; green fluorescence) and either the *cis* (**A**) or *trans* (**B**) Golgi with antibody against GM130 or golgin-97 (Alexa 594; red fluorescence), respectively. Nuclei were stained with DAPI (blue fluorescence) and imaged by deconvolution microscopy. * indicate cells with disassembled Golgi.

Due to Golgi rearrangements observed during FCV [24] and MNV [25] replication, we first determined if the Golgi is morphologically changed during human NV replication. Golgi integrity was examined in Huh7 cells 24 hours post-transfection (hpt) of NV RNA that results in a single cycle of viral replication [30]. The Golgi was examined in cells that expressed the viral capsid protein VP1, which is made late in replication and serves as a marker for cells replicating NV. As evidenced by the elongated, peri-nuclear re-localization of Golgi marker proteins (asterisks, Figure 1A and B), which are characteristic of the disassembled Golgi observed during mitosis [31], [32], the Golgi was disassembled in 53 of 56 (95%) and 42 of 46 (91%) VP1 positive cells based on immunostaining of the *cis* and *trans* Golgi marker proteins GM130 and golgin-97, respectively. This was in contrast to the phenotypically normal, well-compact and condensed Golgi observed in almost all (97%) of non-transfected cells. These results indicate that, like other caliciviruses, NV replication induces disassembly of the Golgi apparatus.

### p22 mediates Golgi disassembly and inhibits protein secretion

**Figure 2:**
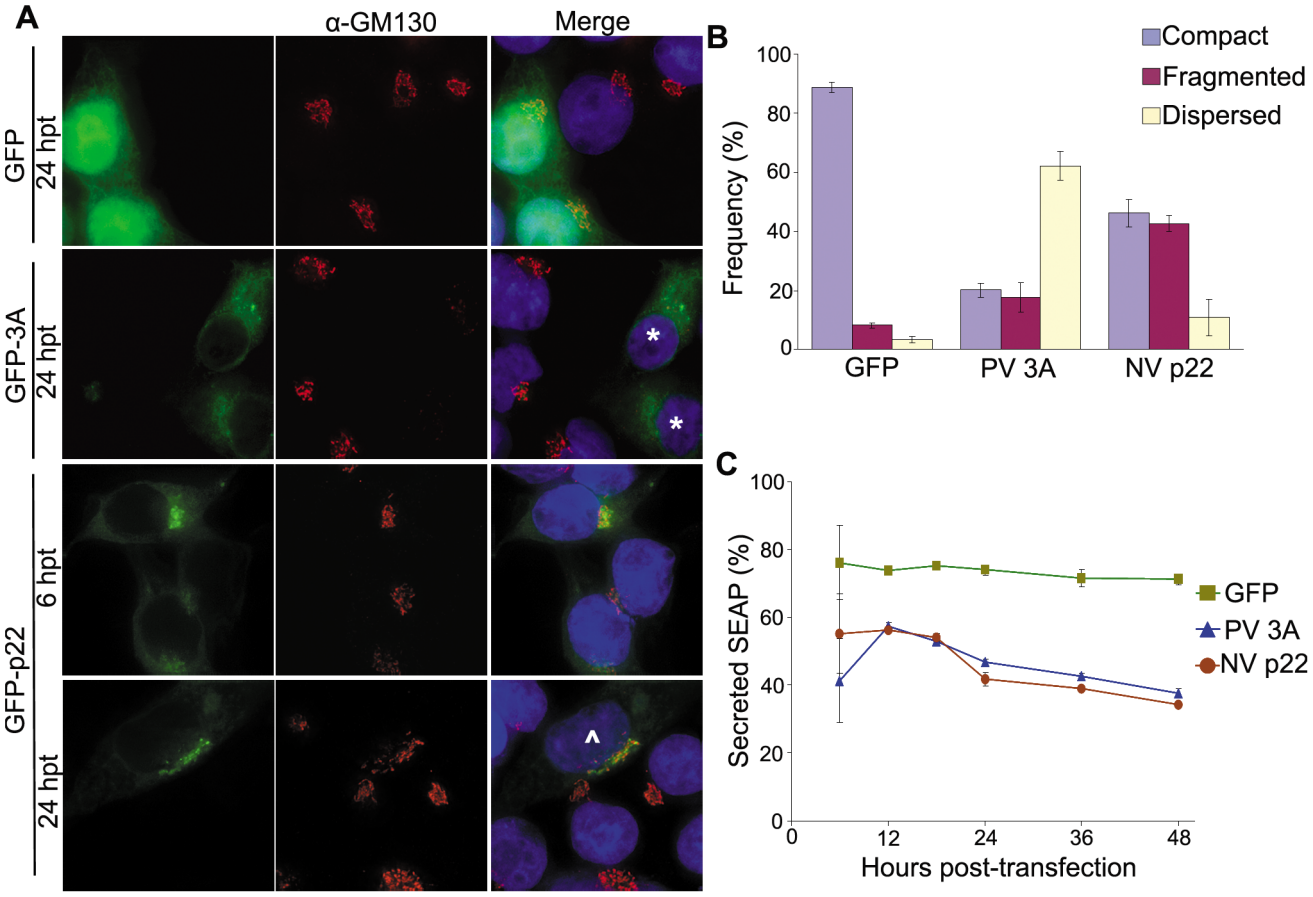
NV p22 induces Golgi disassembly and inhibits protein secretion. (**A**) Cells expressing GFP, GFP-tagged poliovirus (PV) 3A protein, or GFP-tagged Norwalk virus (NV) p22 were stained for the *cis* Golgi marker protein GM130 (Alexa 594-conjugated secondary antibody, red fluorescence) at the indicated times post-transfection. Nuclei were stained with DAPI (blue fluorescence), and cells were imaged by deconvolution microscopy. * indicates cells with dispersed Golgi; ∧ indicates cells with fragmented Golgi. (**B**) Quantitation of Golgi status in cells expressing the indicated proteins at 24 hours post-transfection (n = 3; minimum of 50 cells per experiment; ±SD). Differences between observed phenotypes are detailed in the text. Results are representative of four independent experiments. (**C**) Cells were transfected with the plasmid pCMV-UTR-SEAP expressing GFP, GFP-tagged PV 3A, or GFP-tagged NV p22. Secreted SEAP was quantified (see Materials and Methods section) as a representative indicator of cellular protein secretion at the indicated time points, and was defined by the equation: Secreted SEAP  =  (SEAP_extracellular_/(SEAP_extracellular_ + SEAP_intracellular_)) ×100. Data are representative of three independent experiments (n = 3 for each time point; ±SD).

The NV nonstructural proteins are produced from a self-cleaving polyprotein and are arranged in an order similar to that of the picornavirus polyprotein [33]. p22 is located within this polyprotein in the same location as the picornavirus 3A protein; however, these proteins share no amino acid identity. Although 3A and p22 also differ considerably in molecular weight (10 kDa vs. 22 kDa, respectively) and predicted secondary structure [7% β-sheet and 62% α-helix vs. 13% and 45%, respectively, as predicted by PSIPRID (data not shown)], we hypothesized that p22 contributes to changes in Golgi morpohology during Norwalk virus replication, as is the case for the 3A protein, which induces Golgi disruption in several picornaviruses [28], [34]. To explore this possibility, the subcellular localization of p22 was characterized by expressing p22 with an N terminal GFP tag in 293T cells by transient transfection, and we used an N terminally GFP-tagged poliovirus 3A protein as a positive control. At 6 hpt, p22 localized to the *cis* Golgi (Figure 2A); however, by 24 hpt p22 showed only minimal *cis* Golgi localization and instead was localized immediately adjacent to a phenotypically disassembled Golgi. Similar results were observed when analyzing the *trans* Golgi (Figure S1).

To characterize the Golgi phenotype in cells expressing 3A and p22, we categorized the morphology of the Golgi in cells as either intact (well-condensed and compact Golgi adjacent to the nucleus), fragmented (non-compact and peri-nuclear, but easily detectable Golgi; indicated by carets) or dispersed (extremely diffuse and phenotypically unapparent Golgi; indicated by asterisks), the latter two both constituting disassembled Golgi. In cells expressing GFP alone, Golgi fragmentation and dispersion were only observed in a small subset of cells (Figure 2A and B). In contrast, transient expression of the poliovirus 3A protein (PV 3A) induced significant fragmentation and dispersion of the Golgi in 18% (p = 0.02) and 62% (p = 0.003) of cells, respectively, compared to the Golgi in GFP-transfected cells. Similarly, expression of NV p22 led to a significant increase in the presence of fragmented, though not dispersed, Golgi present in 43% (p = 0.006) and 11% (p = 0.09) of cells, respectively. The significant differences between 3A and p22 in Golgi fragmentation (p<0.01) and dispersion (p<0.003) suggested that the ultimate effect of p22 on Golgi architecture was markedly different from that of 3A, possibly reflecting a different mechanism to induce Golgi disassembly.

We next sought to determine the biological relevance of the observed Golgi structural alterations on cellular protein secretion. To accomplish this, a secreted alkaline phosphatase (SEAP) reporter assay was utilized in which GFP-tagged p22 and SEAP were co-expressed from a di-cistronic vector, pCMV-UTR-SEAP [28]. This vector encodes the gene of interest under a CMV promoter and SEAP, a reporter protein that is rapidly secreted from cells and is a quantitative surrogate of protein secretion [35], which is translated via an internal ribosomal sequence. All proteins expressed using this vector system had N terminal GFP tags, which has been reported to not affect the ability of PV 3A to inhibit protein secretion [12]. At various times post-transfection, media and cell pellets were assayed for extra- and intra-cellular enzymatic SEAP activity, respectively; total SEAP levels did not significantly differ between all proteins expressed at any time point tested. For all constructs, enzymatic SEAP activity was first detectable over background in both fractions at 6 hpt. Expression of GFP alone led to ∼75% secreted SEAP throughout the assay (Figure 2C), whereas expression of PV 3A led to a significant reduction of SEAP secretion (p<0.001) with maximal reduction to 38%, or 53% of GFP alone levels, at the final time point, which is similar to previous results [13], [28], [36]. With similar kinetics to 3A, NV p22 also ultimately inhibited SEAP secretion to 34%, or 48% of GFP alone levels. From this, we concluded that, despite their differing specific effects on Golgi phenotype, NV p22 is able to inhibit SEAP secretion, and therefore cellular protein secretion, to levels similar to PV 3A.

**Figure 3:**
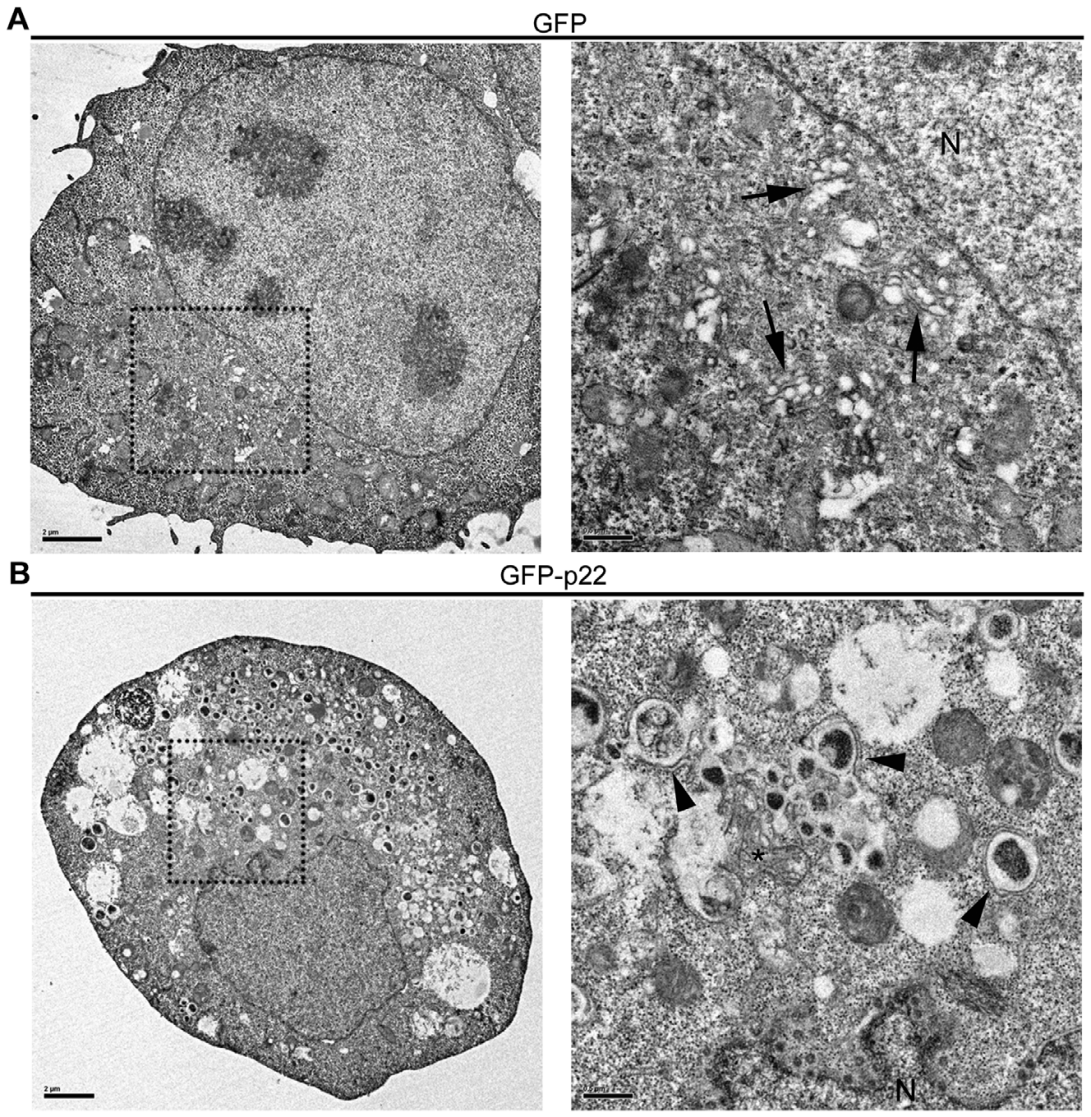
Expression of NV p22 induces alterations in secretory pathway ultrastructure. At 24 hours post-transfection, cells expressing GFP (**A**) or GFP-p22 (**B**) were harvested and flow sorted for expression of GFP. Twenty-four hours after re-plating, cells were fixed and prepared for visualization by electron microscopy. The boxed regions represent the area magnified to the right. Left scale bars represent 2 µm, right scale bars represent 0.5 µm. N =  nucleus; black arrows indicate intact Golgi cisternae; black arrowheads indicate double-membrane vesicles; the asterisk indicates free membranes.

To gain a better understanding of potential ultrastructural alterations induced by p22, cells expressing GFP or GFP-p22 were flow sorted for GFP expression at 24 hpt. After 24 hours of recovery following flow sorting, and therefore 48 hpt, cells were fixed and thin sections were visualized by electron microscopy (EM). After flow sorting, cells expressing GFP alone had intact and peri-nuclearly localized Golgi with cisternal stacks clearly visible in 31 of 59 (52%) cells examined (Figure 3A, arrows). In contrast, cells expressing GFP-p22 had detectable Golgi stacks in just 4 of 57 (7%) cells examined. Instead, GFP-p22 cells exhibited an abundance of large vacuoles, loose single membranes (Figure 3B, asterick), and double-membrane structures (Figure 3B, arrowheads). Many of these structures had what appeared to be cargo inside them, but the nature of this cargo was unclear as these structures were much larger than would be expected for those containing normal secretory pathway cargo. These results confirmed the immunofluorescence observations of a disassembled and phenotypically abnormal Golgi, demonstrating that expression of p22 led to rearrangements and alterations to various components of the secretory pathway, as would be expected during antagonism of this pathway.

### Amino acids 50–148 mediate Golgi localization of p22

**Figure 4:**
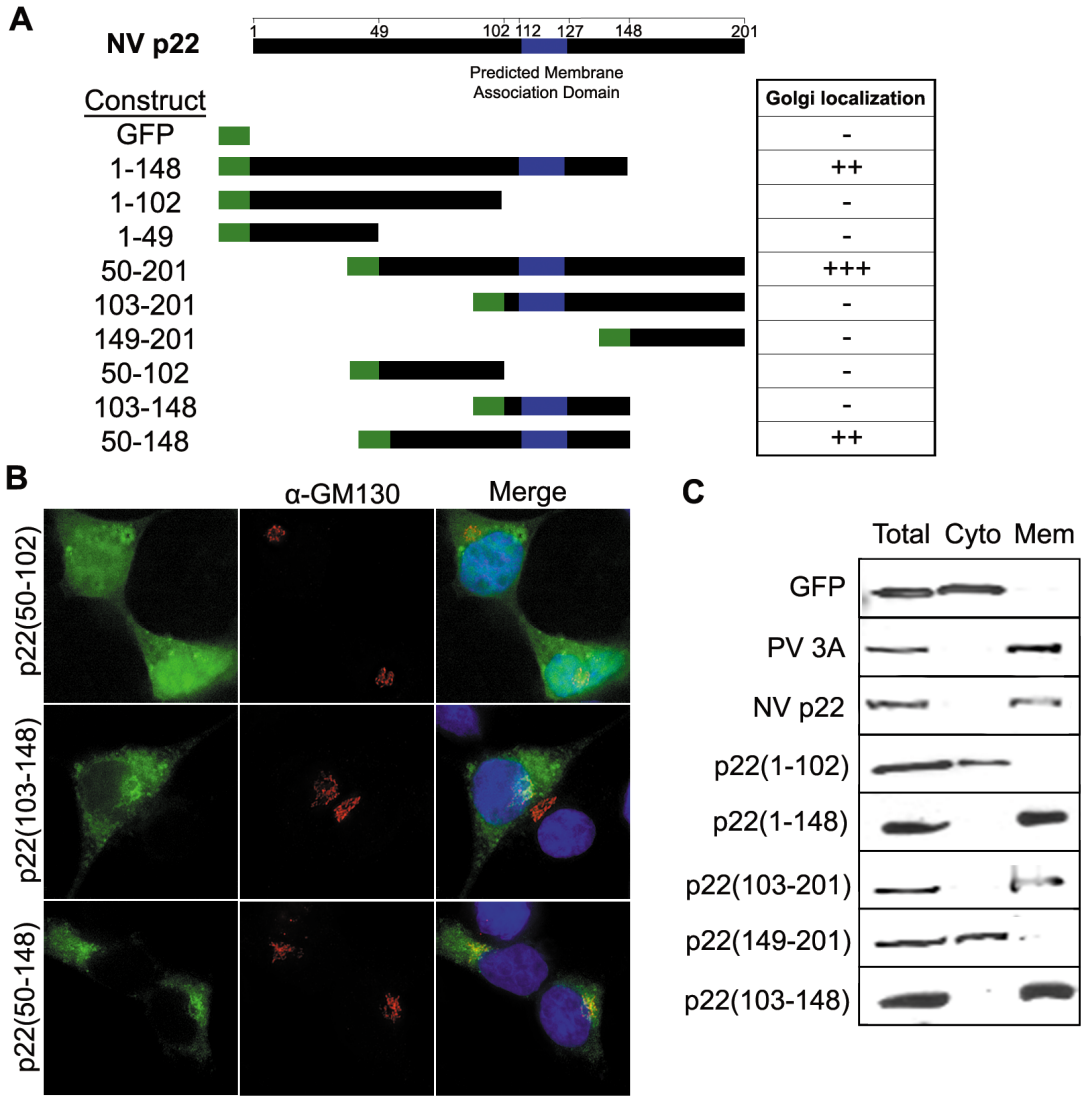
Amino acids 50–148 are sufficient to mediate Golgi localization of NV p22. (**A**) GFP tagged N and C terminal deletion mutants of p22 were generated and, following expression in cells for 24 hours, were scored for their ability to localize to the *cis* Golgi. Amino acid numbering corresponds to NV sequence (NC_001959). (**B**) Cells expressing GFP-tagged p22(50–102), p22(103–148), and p22(50–148) were immuno-stained with antibody against the *cis* Golgi marker protein GM130 (Alexa 594-conjugated secondary antibody, red fluorescence), stained with DAPI (blue fluorescence), and imaged by deconvolution microscopy. (**C**) Cells expressing GFP, GFP-tagged PV 3A, GFP-tagged NV p22, or the indicated deletion mutants of p22 were harvested at 24 hpt. Cytosolic and membranous fractions of cells were collected and proteins were detected by western blot with monoclonal antibody against GFP.

We next determined which regions of p22 are responsible for Golgi localization and/or fragmentation. N and C terminal deletion mutants of p22 with N-terminal GFP tags were generated (Figure 4A) with respect to predicted α-helices and β-sheets. GFP-tagged deletion mutants encoding amino acids 50–148 localized to the Golgi (summarized in Figure 4A), whereas constructs that lacked either amino acids 50–102 or 103–148 failed to specifically localize to the Golgi; none of the deletion mutants tested were able to induce Golgi disassembly equivalent to wildtype p22. Expression of amino acids 50–102 localized non-specifically throughout cells (Figure 4B), in contrast to amino acids 103–148, which predominantly exhibited a reticular pattern throughout the cytoplasm suggestive of general intracellular membrane localization. When these regions were expressed together as p22(50–148), the protein was predominantly Golgi localized and the Golgi was phenotypically intact.

We next examined the factors between amino acids 50–148 that mediate the subcellular localization of p22. Computational analyses of full-length p22 with PHDhtm (available online at www.predictprotein.org [37]) predicted an amphipathic α-helical transmembrane (TM) domain between amino acids 112 and 127. A membrane association domain (MAD) within p22 would provide further similarity to the picornavirus 3A protein, which encodes an amphipathic α-helix that acts as both a TM domain and a MAD at the C terminus of the protein [38]. The localization of p22(103–148) and p22(50–148) compared to p22(50–102) (Figure 4B) supported the hypothesis that p22 localizes to the Golgi in part due to membrane association contributed by this domain.

To determine if amino acids 103–148 mediate membrane association of p22, we expressed GFP alone, GFP tagged 3A or p22, or various deletion mutants of p22 in 293T cells and isolated cytosolic and membranous fractions. As expected, GFP was present solely in the cytosolic fraction and PV 3A solely in the membranous fraction of cells (Figure 4C). p22 was also present in the membranous fraction, confirming that it is a membrane-associated protein. Only the p22 deletion mutants that contained amino acids 103–148 were present in the membranous fraction of cells, including a construct encoding amino acids 103–148 alone; all constructs that did not contain amino acids 103–148 were present in the cytosolic fraction. This indicated that residues 103–148 are responsible for membrane association of p22, likely due to an amphipathic α-helix between amino acids 112–127.

### p22 contains an ER export signal mimic that is highly conserved in human noroviruses

**Figure 5:**
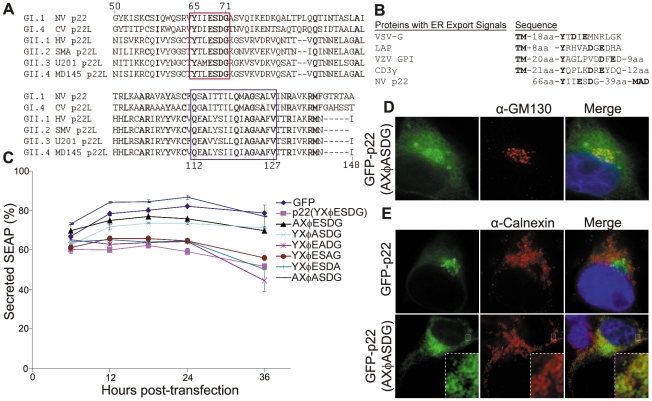
A conserved noroviral ER export signal is necessary for p22 to inhibit protein secretion. (**A**) Amino acids 50–148 from NV p22 were aligned with homologues [“p22-like (p22L) proteins”] from representative genogroup 1 (GI) and genogroup 2 (GII) human noroviruses of various genotypes. The figure illustrates six of 72 sequences analyzed. Conserved residues are shown in bold; the blue box indicates conservation of the membrane association domain (MAD); the red box indicates conservation of the YXΦESDG motif. NV is Norwalk virus (NC_001959), CV is Chiba virus (AB042808), HV is Hawaii virus (U07611), SMV is Snow Mountain virus (AY134748), U201 is Saitama U201 virus (AB039782), and MD145 is MD145 virus (AY032605). (**B**) Alignment of NV p22 with various cellular and viral proteins that contain an ER export signal. VSV G is the vesicular stomatitis virus glycoprotein, LAP is lysosomal acid phosphatase, VZV GPI is varicella zoster virus glycoprotein I, and CD3γ is a component of the T cell receptor. Adapted from Nishimura and Balch, 1997 [9]. (**C**) Cells were transfected with the plasmid pCMV-UTR-SEAP expressing GFP, GFP-tagged NV p22, or the indicated mutants within the ER export signal of p22. Secreted SEAP was quantified (see Materials and Methods) as a representative indicator of cellular protein secretion at the indicated time points and was defined by the equation: Secreted SEAP  =  (SEAP_extracellular_)/(SEAP_extracellular_ + SEAP_intracellular_) ×100. Data are representative of three independent experiments (n = 3 for each time point; ±SD). (**D** and **E**) p22 with the YXΦESDG motif mutated to AXΦASDG (**D**; **E**, bottom panels) or wildtype NV p22 (**E**, top panels) were expressed as GFP fusion proteins. Cells were fixed at 24 hpt and stained with antibody against the *cis* Golgi marker protein GM130 (**D**) or the endoplasmic reticulum marker protein calnexin (**E**) (Alexa 594-conjugated secondary antibody, red fluorescence). Nuclei were stained with DAPI (blue fluorescence) and cells were imaged by deconvolution microscopy. The inset in **E** is an 8X magnification of the boxed region.

Because amino acids 103–148 alone resulted in membrane, but not Golgi, localization, we next examined the sequence between amino acids 50–102 for additional factors that might facilitate Golgi localization of p22. A multiple sequence alignment of p22 and its homologues from the 72 available full-length human norovirus sequences from viruses classified in different genogroups (GI.1 to GII.12) found 13% amino acid identity between all proteins (summarized in Figure 5A). This defined p22 as the most variable protein in human noroviruses [39]. Despite this, two regions within amino acids 50–148 showed clear sequence conservation. The first region was the MAD (Figure 5A, blue box), indicating that this domain is well-conserved amongst homologues of p22 and likely serves a similar function between genogroups. The second was a YXΦESDG motif (Figure 5A, red box), where X is any amino acid and Φ is a bulky, hydrophobic residue (e.g. M, I or L), which was fully conserved in 65 of the 72 (90%) sequences available for examination (summarized in Figure 5A). Unexpectedly, conservation of this motif was limited to p22 homologues of human noroviruses. It was not present in either the MNV or FCV homologues of p22, both of which exhibit very low amino acid identity with NV p22, and was similarly absent in the 3A protein of poliovirus and coxsackievirus B3. Further examination revealed that the YXΦESDG motif resembled the criteria for a di-acidic ER export signal, which typically contains a YXXΦ motif immediately proximal to two acidic residues separated by a single amino acid [e.g. YXXI(E/D)X(E/D)], all of which are located on the cytosolic side of a TM domain (Figure 5B) [9], [10]. Such signals increase the rate of protein export from the ER into COPII vesicles and onward to the Golgi [9], [10], [40].

To explore if this conserved norovirus motif could play a role in the previously demonstrated inhibition of cellular protein secretion by p22 (Figure 2C), individual alanine mutations were made within each of the conserved residues within the putative ER export signal of p22 and tested as before using the SEAP system. Mutations within the S, D and G residues had no effect on the ability of p22 to inhibit cellular protein secretion (Figure 5C); however, mutation of both the Y and E residues led to intermediate levels of protein secretion. When these two residues were combined into a single AXΦASDG construct, SEAP secretion at 36 hpt was not statistically different from that of GFP alone (p = 0.08). Total SEAP expressed by all p22 mutants did not significantly differ from that of wildtype p22 and similar levels of all p22 proteins were expressed, as confirmed by western blot analysis of intracellular fractions at 36 hpt (Figure S2), showing that the observed decreases in inhibition of protein secretion were not due to changes in protein expression or stability.

To examine Golgi phenotype in cells expressing the AXΦASDG mutant of p22, we next explored the phenotype of the Golgi in cells expressing this construct. By electron microscopy at 48 hpt and after flow sorting, cells expressing p22(AXΦASDG) had wildtype Golgi (data not shown) with intact cisternae present in 26 of 56 (46%) cells, similar to the frequency of intact Golgi in the presence of GFP alone. Consistent with this observation and the inability of p22(AXΦASDG) to inhibit SEAP secretion, the Golgi was intact in cells expressing p22(AXΦASDG) by immunofluorescence at 24 hpt (Figure 5D). Moreover, the mutant was much more diffuse throughout the cytoplasm of cells with few to no puncta, and exhibited a prominent reticular pattern suggestive of ER localization. To confirm this observation, we next stained cells expressing wildtype p22 or p22(AXΦASDG) for the ER marker protein calnexin, the use of which was validated by complete co-localization with protein disulfide isomerase (Figure S3), another ER marker protein.

Wildtype p22 did not co-localize with the ER-marker protein calnexin (Figure 5E), as would be expected for a protein that utilizes an ER export signal [40], [41]. In contrast, the AXΦASDG construct of p22 exhibited a more diffuse and reticular staining pattern, much of which overlapped with the ER (Figure 5E, inset), suggesting that the mutated residues were either re-localizing the protein to the ER or slowing the trafficking of the protein from the ER. Constructs that had individual mutations within the Y and E residues had intermediate ER localization, and a construct that had both the glutamic and aspartic acid residues mutated to alanine exhibited ER localization and SEAP secretion equivalent to that of the YXΦASDG construct (data not shown). These data indicate that the Y and E residues within the conserved YXΦESDG motif are necessary for the proper localization of p22 and may constitute a unique arrangement of an ER export signal.

Taken together, these data demonstrate that despite altered trafficking within the secretory pathway, p22(AXΦASDG) that was not ER-localized appeared to be retained at the Golgi, reflecting the ability of ER-localized proteins to be non-specifically trafficked from the ER to the Golgi in the absence of a specific export signal [9], [10], [40]. This indicates that the Y and E residues of the YXΦESDG motif together are critical for the cellular localization of p22, as well as inhibition of protein secretion and Golgi disassembly.

**Figure 6:**
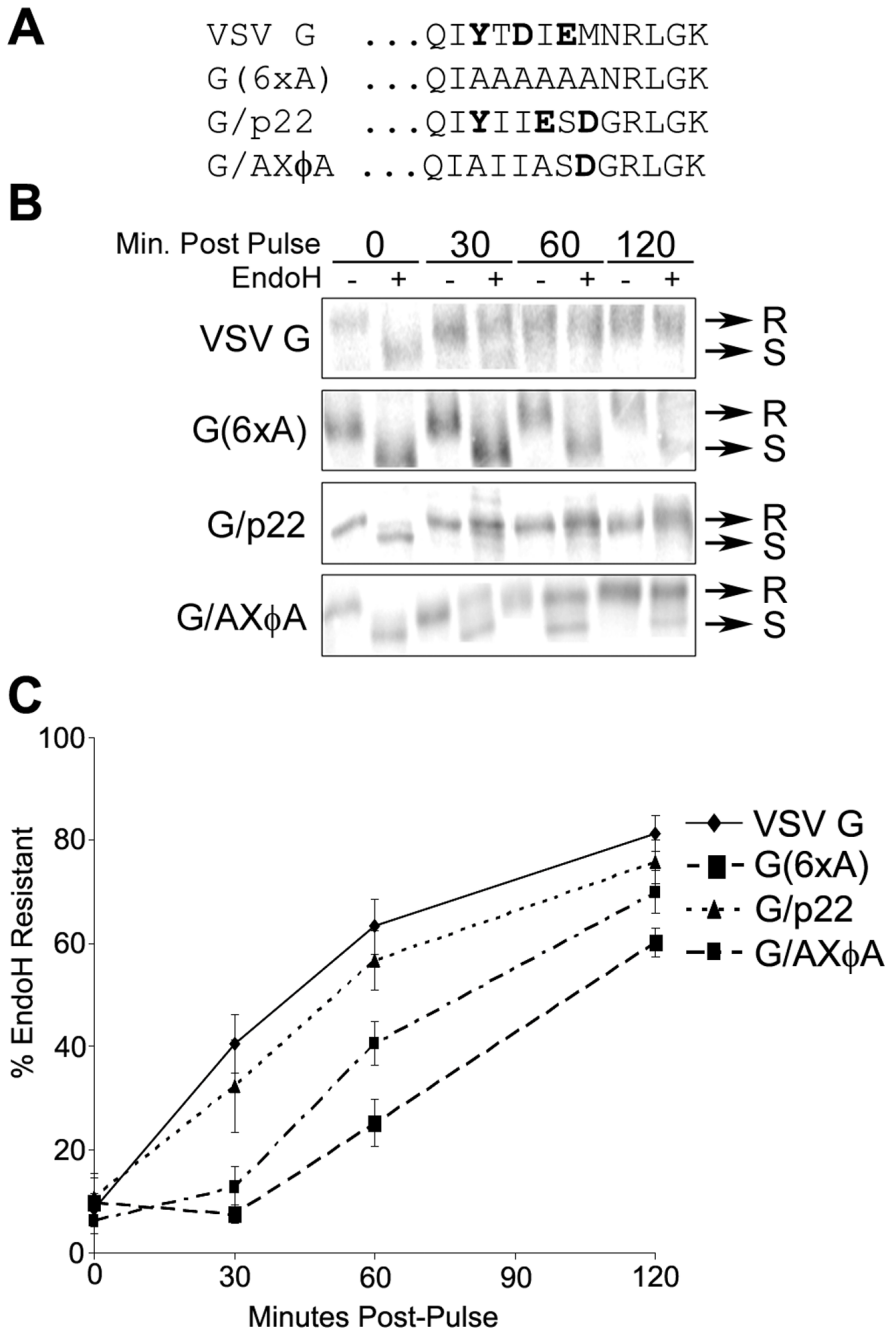
The ER export signal mimic of p22 can substitute for the signal of VSV G. (**A**) Summary of the sequence of the wildtype, mutant, and chimeric VSV G proteins used. Critical residues of the VSV G ER export signal and homologous regions of p22 are shown in bold. (**B**) Representative individual samples from EndoH sensitivity assay of VSV G proteins. Wildtype (G), mutant [G(6xA)] and chimeric (G/p22 and G/AXΦA) VSV G proteins were metabolically labeled with ^35^S-Methionine at 24 hours post-transfection and incubated for the indicated period of time, harvested in lysis buffer, immuno-precipitated with monoclonal antibody against the luminal domain of VSV G and digested with endoglycosidase H (EndoH). R =  EndoH resistant; S =  EndoH sensitive. (**C**) Cells were transfected with plasmids encoding the indicated constructs of VSV G. At 24 hours-post-transfection, cells were labeled with ^35^S-Met and at various times post-pulse cells were harvested, immuno-precipitated with antibody against the luminal domain of VSV G and assayed for their sensitivity to EndoH. Data are composite (mean ± SD) of six individual samples (n = 6) for each time point from two independent experiments.

Although the conserved YXΦESDG motif was clearly critical to the cellular effects exhibited by p22, two aspects of this signal remained unclear: 1) if this motif is indeed a functional ER export signal; and 2) if this motif alone is sufficient to antagonize ER/Golgi trafficking. To answer both of these questions, we next directly tested if the conserved motif within p22 could functionally substitute for a *bona fide* ER export signal. To do this, the well-characterized ER export signal within the vesicular stomatitis virus glycoprotein (VSV G) was replaced with either the conserved motif found within p22 (G/p22), the mutated p22 signal that abrogates the downstream effects of p22(G/AXΦA), or a construct of VSV G that has the entire ER export signal mutated to all alanines (G/6xA) and has a significantly decreased rate of ER export [40] (Figure 6A). The efficiency of ER export of VSV G or the various chimeric or mutant G proteins was examined by a previously described technique [9], [10], [40], [42] in which newly made protein is metabolically labeled with ^35^S-methionine. At the indicated time points, G is purified and digested with EndoH, which only digests G that has not yet reached the Golgi, resulting in a shift in its apparent molecular weight following resolution by SDS-PAGE (Figure 6B). The percent of G that is resistant to EndoH allows quantitation of the amount of protein that is ER-associated compared to protein that had reached the Golgi, thereby yielding a direct measure of ER export efficiency.

As expected, wildtype VSV G rapidly became resistant to EndoH digestion, whereas G(6xA) exhibited a significant decrease in kinetics of ER export compared to wildtype G [p<0.00001 for all time points except 0 minutes post-pulse (mpp)] (Figure 6C). In contrast, G/p22 demonstrated ER export statistically indistinguishable from wildtype VSV G (p≥0.07 for all time points except 0 mpp), indicating that the conserved motif found within p22 can substitute for the signal found within G and is therefore a functional ER export signal. In contrast, and similar to G(6xA), G/AXΦA had significantly decreased kinetics of ER export (p≤0.001 for all time points except 0 mpp). The ability of G/AXΦA to ultimately become resistant to EndoH digestion more efficiently than G(6xA) is likely attributable to the presence of the aspartic acid residue on G/AXΦA, which has previously been shown to improve ER export efficiency compared to alanines alone [9], [10], [40]. In addition, the Golgi was phenotypically intact in cells expressing both wildtype G and G/p22 (data not shown), suggesting that protein trafficking is not inhibited due solely to the presence of the YXΦESDG motif.

Taken together, these data suggest that the motif found within p22 can function for the well-characterized ER export signal found within VSV G and thus this motif constitutes a true ER export signal. For this reason, we henceforth refer to the YXΦESDG motif within p22 as a mimic of an ER export signal, or MERES, motif. In addition, mutation of two residues within the MERES motif that are critical to the function of p22 is sufficient to decrease ER export efficiency. This observation coupled with the inability of the MERES motif alone to induce Golgi disassembly when expressed on VSV G demonstrated that it alone is insufficient to antagonize ER/Golgi trafficking.

### p22 targets COPII, but not COPI, trafficking

We lastly sought to determine, first, what is the fate of the cargo in the secretory pathway? And second, does p22 target the anterograde or retrograde pathway of ER/Golgi trafficking? To answer both of these questions, we utilized the SEAP vector described above to monitor the localization of COPI and COPII vesicle marker proteins with respect to secretory pathway cargo, in this case again monitoring SEAP itself as the cargo, and alterations that occur due to the presence of p22.

**Figure 7:**
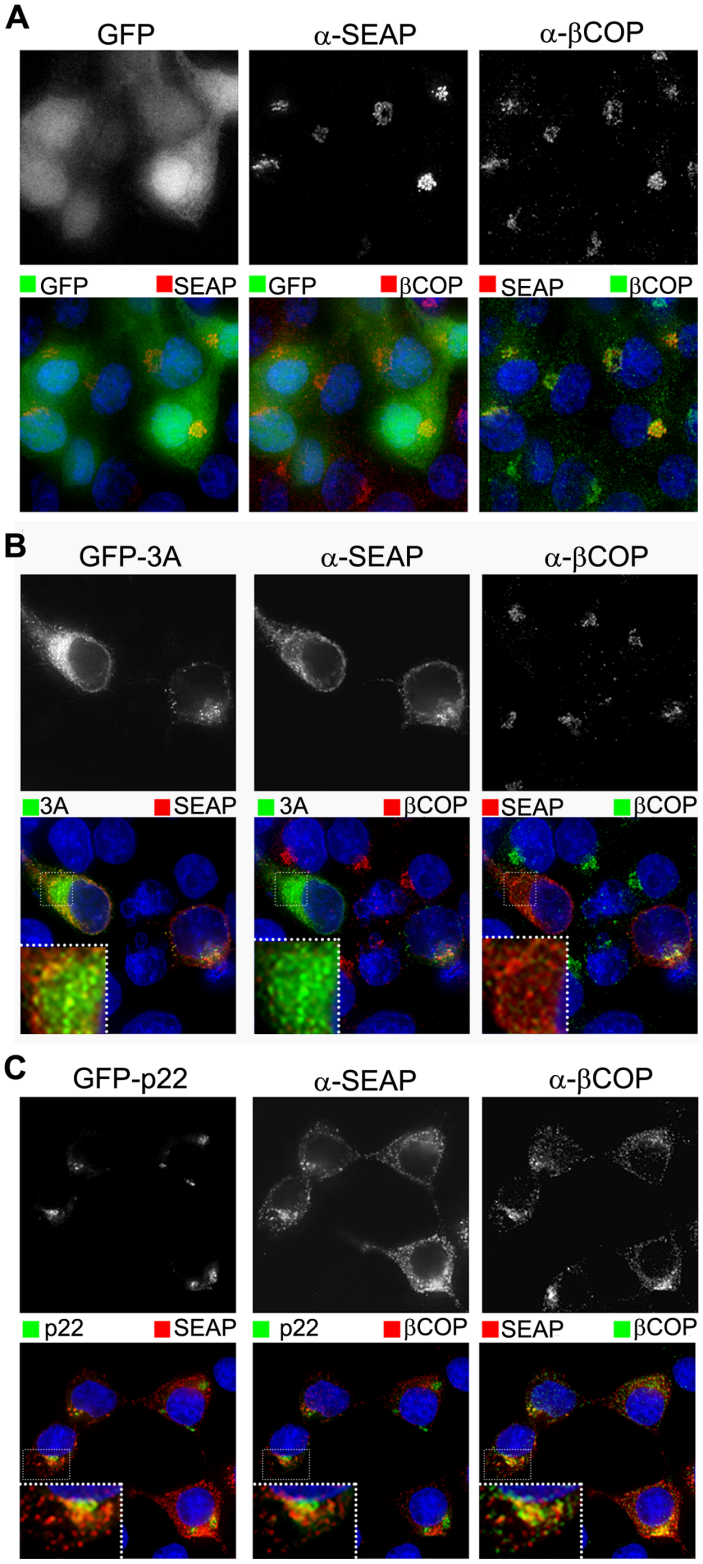
SEAP is differentially retained in the presence of PV 3A and NV p22. Cells expressing GFP alone (**A**), or GFP-tagged 3A (**B**) or NV p22 (**C**) were fixed at 24 hpt and immuno-stainined with antibody against SEAP (Alexa 594-conjugated secondary antibody) and the COPI marker protein β-COP (Alexa 647-conjugated secondary antibody), stained with DAPI (blue fluorescence), and imaged by deconvolution microscopy. Channels were pseudo-colored as indicated for merged images. Insets in **B** and **C** represent a 6X zoom of the boxed region.

The 3A protein from several picornaviruses inhibits protein secretion by deregulating COPI vesicle budding from the *cis* Golgi [12], [13], [43]. To determine if p22 might utilize a similar mechanism of action, the phenotype of COPI vesicle transport in cells expressing p22 was explored by immunofluoresence of the COPI marker protein β-COP. In the presence of GFP alone, SEAP was present only in an intact Golgi, where COPI puncta are prominently, though not exclusively, localized (Figure 7A). As expected, SEAP was prominently retained in cells expressing both PV 3A (Figure 7B) and NV p22 (Figure 7C), although with different phenotypic localizations. In 3A-expressing cells, COPI puncta were diffuse and unapparent, and SEAP was retained in diffuse, minimally punctate cellular structures reminiscent of Golgi that has been redistributed into the ER due to antagonism of trafficking by 3A, as has been reported previously [12], [34], [44]. In contrast, COPI puncta in cells expressing p22 were apparent but re-localized widely throughout the cytoplasm, demonstrating a failure of these vesicles to properly localize and/or traffic within cells. SEAP in p22-positive cells was present both in discrete punctate vesicles that did not co-localize with COPI puncta, and also in peri-Golgi clusters that did co-localize with COPI puncta. This suggested that SEAP, and therefore cellular cargo, was being retained in non-COPI puncta and at a *cis* Golgi site due to the expression of p22. This suggested that the retrograde, Golgi-to-ER arm of the secretory pathway was intact, but an aspect of the forward pathway was non-functional in p22-expressing cells. These data demonstrate that, in contrast to 3A, p22 does not specifically target COPI trafficking, but does induce cellular cargo retention between the ER and Golgi.

**Figure 8:**
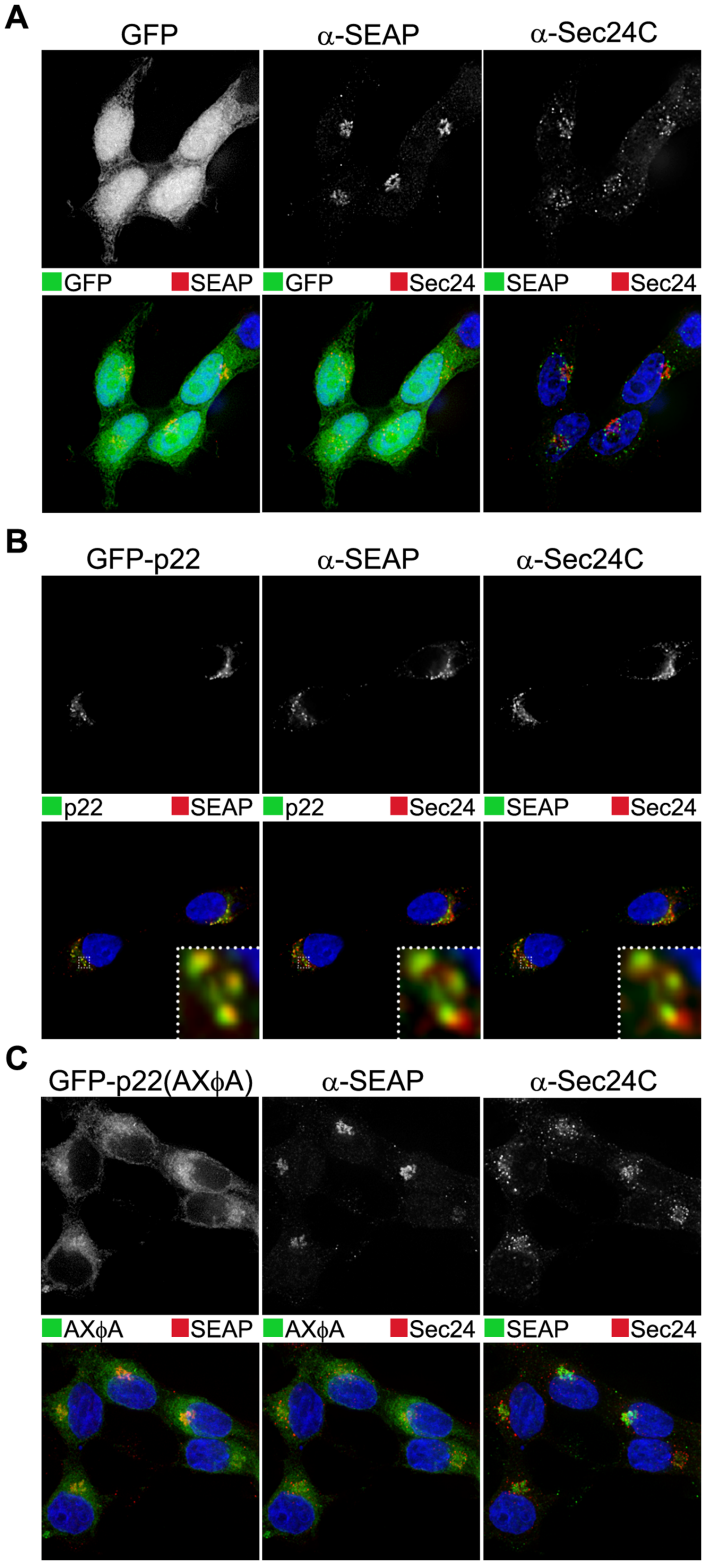
Secretory pathway cargo is retained in COPII vesicles in the presence of p22, but not p22(AXΦA). Cells expressing GFP alone (**A**), or GFP-tagged NV p22 (**B**) or p22(AXΦA) (**C**) were fixed at 24 hpt and immuno-stained with antibody against SEAP (Alexa 594-conjugated secondary antibody) and the COPII marker protein Sec24C (Alexa 647-conjugated secondary antibody), stained with DAPI (blue fluorescence), and imaged by deconvolution microscopy. Channels were pseudo-colored as indicated for merged images. Inset in **B** represents an 8X zoom of the boxed region.

Due to these observations and because ER export signals promote the rapid and direct uptake of cargo into COPII-coated vesicles, which are necessary for ER-to-Golgi protein trafficking, we hypothesized that p22 is acting on the forward, ER-to-Golgi trafficking pathway to inhibit COPII vesicle budding or trafficking to the Golgi to thereby induce Golgi disassembly and inhibit protein secretion. To test this hypothesis, we again examined the specific sub-cellular localization of the retained SEAP that was present in discrete cytoplasmic puncta. Under the same di-cistronic SEAP expression system, in the presence of GFP alone SEAP was again localized exclusively in a phenotypically intact Golgi with COPII puncta immediately surrounding it, although with a near complete lack of co-localization with COPII puncta (Figure 8A). In contrast, in the presence of p22 SEAP was localized in peri-nuclear puncta (Figure 8B), similar to the phenotype of a disassembled Golgi previously described (Figure 2A). Both SEAP and p22 also co-localized with COPII puncta that were also prominently re-localized to the same presumably peri-Golgi structures, although there was some diffuse, likely ER-localized, COPII staining that did not localize with either p22 or SEAP. Additionally, these discrete p22-positive puncta co-localized with SEAP and COPII vesicles (Figure 8B, inset). This suggested that p22 and SEAP are retained with COPII puncta that have properly budded from the ER, but have been mislocalized and did not properly traffic into the Golgi. Due to the apparent size of the p22/SEAP/COPII puncta observed by immuno-fluorescence, it is tempting to speculate that the cellular vesicles with cargo inside them that were observed by EM (Figure 3B) may be enlarged COPII puncta that were mislocalized within cells. When the AXΦA mutant of p22 was expressed, SEAP and COPII puncta both returned to a wild type distribution (Figure 8C) that was phenotypically indistinguishable from cells expressing GFP alone. This demonstrated that, in the presence of p22 and dependent upon the MERES motif, both COPII puncta and their cargo are mislocalized, suggestive of a failure of vesicles to either traffic to or fuse with the Golgi apparatus.

## Discussion

We have described for the first time a novel function for the Norwalk virus nonstructural protein p22. The major new findings of this study can be summarized as follows: 1) independent expression of p22 disassembles the Golgi, which also occurs during NV replication, and inhibits cellular protein secretion; 2) subcellular localization of p22 depends on a motif that mimics a cellular ER export signal in both sequence and function, which we have named a MERES motif; and 3) p22 depends on the MERES motif likely to antagonize COPII vesicle trafficking, resulting in Golgi disassembly and an inhibition of cellular protein secretion. Due to the observed trafficking of p22, we propose that p22 is initially localized to the membranes of the secretory pathway via amino acids 103–148, which contain a membrane association domain. The MERES motif then mediates the uptake of p22 onto COPII vesicles, which mislocalize within cells and inhibit, by an as-of-yet undetermined mechanism, proper trafficking to or fusion with the Golgi. Restricting the proper flow of COPII vesicles then induces disassembly of the Golgi and ultimately results in an inhibition of cellular protein secretion.

The MERES motif found within p22 is somewhat unconventional for an ER export signal in that p22 does not encode a signal sequence to mediate ER import, is not glycosylated, and is not exclusively ER localized at any point during expression (Figure 4D and data not shown). Moreover, it has several unique features compared to other known di-acidic ER export signals. First, instead of the tyrosine residue being located between the MAD and the acidic residues, it is located N terminal to the acidic residues and MAD. Second, instead of encoding two amino acids between the Y and Φ residues, p22 encodes only a single residue. Third, most, though not all, ER export signals are located towards the extreme C terminus of proteins, whereas the signal found within p22 is located in the middle of the protein. The human asialoglycoprotein receptor H1 subunit is the only other protein described to have this arrangement of a di-acidic ER export signal [9]. Additionally, we have no data to support any portion of p22 being present inside the lumen of the ER and instead p22 appears to be a peripheral membrane protein based on sodium carbonate extraction (data not shown). For these reasons, the YXΦESDG motif found within p22, though similar in sequence and function to a traditional di-acidic ER export signal, may in reality simply mimic the function of these signals to exploit the cellular machinery responsible for protein secretion in order to recruit p22 to COPII vesicles. This lends support to the nomenclature of this motif being a mimic of an ER export signal, as it shares many of the features and effects of a traditional ER export signal, but ultimately has a unique composition and function.

Furthermore, p22 seems to depend on the MERES motif to function as a secretory pathway inhibitor; however this motif alone is not responsible for antagonism, as replacement of the ER export signal from VSV G with the MERES motif functionally restored ER export efficiency without any obvious impediment. In support of this, none of the deletion mutants of p22 induced Golgi disassembly, although amino acids 50–148 were sufficient to mediate Golgi localization. Similarly, these same residues were sufficient to mediate COPII localization, but do not lead to the mislocalization of COPII puncta (data not shown) that was observed for full length p22. Taken together, these data suggest that the MERES motif of p22 alone is necessary but not sufficient to inhibit the secretory pathway. A second as-of-yet uncharacterized factor, contained within or dependent on the N and/or C termini based on deletion mutant experimentation, is required as a subsequent step in secretory pathway antagonism. This idea is substantiated by chimeric G/p22 properly trafficking to the Golgi and becoming resistant to Endo H digestion with kinetics equivalent to wildtype G. Possibilities for the function of this second factor are many, including binding to and/or disrupting a cellular tethering protein [45], [46] or a member of the p24 family that covers COPII vesicles [47], or antagonizing SNARE-mediated vesicle fusion by interaction with a regulatory protein such as VAP-A/B [48], all of which are critical components of ER/Golgi trafficking. This latter possibility is especially attractive, as VAP-B has previously been demonstrated to be a binding partner for another Norwalk virus nonstructural protein [49], and thus may be an over-arching target of the secretory pathway by noroviruses. Both of these possibilities are currently under investigation as potential targets for p22 and as scaffolding factors upon which noroviruses may anchor their genome replication.

The data presented in this study support, but do not prove, that p22 targets COPII vesicle trafficking; this is further complicated by the examination of cells by immunofluorescence primarily at 24 hpt, which may not reflect steady-state localization. Proving this mechanism will require demonstrating a specific binding to or inhibition of the trafficking of COPII vesicles using *in vitro* assays. Unfortunately, these studies have not been possible due to the inability of producing purified p22 for such studies as well as the lack of an antibody to p22 that allows for immunoprecipitation assays. Therefore, until a direct interaction is demonstrated, it remains possible that p22 could target a non-COPII aspect of the secretory pathway to mediate inhibition. p22 does not activate Arf1 or Sar1 (data not shown), making targeting of the formation of COPI vesicles in the same manner as PV and CVB3 3A, or COPII vesicles by a unique approach, unlikely. A *trans* Golgi mechanism of action seems similarly unlikely based on lack of localization of wildtype p22 with the trans Golgi marker protein golgin 97 (Figure S2); the same is also true for possible targeting of endosomes by p22 (data not shown). Thus, although there are many possible alternative explanations for the observed effects of p22, specific targeting and mislocalization of COPII vesicles is at present the most likely explanation.

Although no other cellular or microbial protein to date has been described to use the arrangement of a MERES motif that p22 employs to inhibit the secretory pathway, several previously characterized secretory pathway antagonists have potential ER export signals or mimics thereof. The *Eschericia coli* protein NleA inhibits COPII-dependent export from the ER by direct interaction with Sec24 [50], and the cellular proteins STAM-1 and -2, which are involved in the signaling of growth factors and cytokines, regulate Golgi architecture by interaction the Sec13/31 COPII cage components [51]. Examination of the primary amino acid sequence of NleA and STAM-1/2 revealed motifs similar to a di-acidic ER export signal (unpublished observation). If further studies determine these motifs directly contribute to either the cellular localization or, for STAM-1/2, proper ER export, this would provide support for the idea that ER export signals or their mimics can be used not only to facilitate ER export, but also to promote interaction with COPII vesicles to mediate specific antagonism of the secretory pathway.

Both similarities and differences were noted between p22 and the picornavirus 3A protein. Both proteins localize to membranes via an amphipathic alpha helix, and both inhibit ER-to-Golgi trafficking to decrease cellular protein secretion. However, the mechanism of this shutoff appears to be quite distinct between Norwalk virus and picornaviruses. Inhibition of protein secretion and Golgi disassembly are in some cases separable and distinct, as is the case for PV infection [44], whereas in other cases one will follow the other, for example during cell division [31], [52]. There were also clear ultrastructural similarities between cells expressing p22 and 3A in inducing the accumulation of free membranes, double-membrane vesicles and vacuoles [12], [34], although cells expressing p22 did not exhibit the swelling of the ER reported for PV 3A [34] or the crystalloid ER patterns seen after expression of the hepatitis A virus 2C and 2BC proteins that also induce significant membrane rearrangements [53]. This further supports the similarity of p22 and 3A in secretory pathway antagonism, but through different arms of this pathway. NV p22 therefore may be more similar in the cellular effects of the hepatitis C virus NS4A/B protein, which, though less studied than PV 3A, antagonizes ER-to-Golgi trafficking, and induces the accumulation of “membranous webs,” vacuoles and double-membrane vesicles, but not ER swelling [54].

Although all the effects of NV infection on the secretory pathway have not yet been explored, the results presented here demonstrate that, like PV 3A, direct antagonism of the secretory pathway is a cause, not an effect, of Golgi disassembly by p22. However, whereas at least two picornavirus 3A proteins interact with the cellular protein GBF-1 to inhibit COPI vesicle budding [13], NV p22 instead appears to target COPII vesicles with dependence upon a motif that is absent from picornavirus 3A proteins, suggesting that these two proteins take different approaches to the same ultimate outcome. It therefore appears that, like several picornaviruses [28], [29], noroviruses encode two proteins, p22 and p48 (this study and [27]), with redundant functions of antagonizing the secretory pathway.

FCV p30, a homologue of p22, lacks an ER export signal and localizes exclusively to the ER when independently expressed ([26] and our unpublished observation). This is in line with the inability of BFA to inhibit FCV replication [24], as it does several picornaviruses, and further supports the notion that caliciviruses utilize a different architecture of cellular machinery for replication than do picornaviruses. A recent study has shown that MNV is also resistant to the cellular effects of BFA and does not have an effect on gross Golgi morphology [55]. It will therefore be interesting to determine the roles of p22 homologues from MNV and FCV in possible antagonism of ER/Golgi trafficking, as this would shed much light on similarities and differences between animal and human caliciviruses.

The biological significance of antagonism of the secretory pathway by p22 remains to be understood. This may facilitate viral pathogenesis rather than replication in a manner similar to the effect of the picornavirus 3A proteins that inhibit the immune response to virus-infected cells [14]–[16], ultimately leading to a more pathogenic infection [12]. Future study of the immune response to norovirus infection should consider secretory pathway antagonism by p22, as this protein may be key in deactivating interferon (IFN) and/or cytokine signaling following infection. Additionally, analysis of the cellular response to NV infection has demonstrated that NV is sensitive to IFN when exogenously added to cells replicating the NV genome [56], [57]; however, NV does not induce the IFN pathway or IRF3 activation in Huh7 cells that support a single round of virus replication [58]. Although p22 may be contributing to a reduction in IFN release from cells, the possibility that p22 has additional inhibitory effects on the IFN pathway remains to be explored.

Since viruses utilize cellular processes and machinery for replication, understanding the mechanisms by which viruses parasitize the cell will both increase our understanding of these pathways and aid in the design of effective anti-viral countermeasures. The human norovirus nonstructural protein p22 encodes a novel and well-conserved motif that mimics a traditional di-acidic ER export signal. Instead of increasing the rate of protein trafficking in the secretory pathway, as is the normal function of these signals, the ER export signal mimic allows p22 to gain access to the secretory pathway, induce Golgi disassembly and inhibit cellular protein secretion. This is the first instance in which a pathogen has been described to use a motif similar to an ER export signal to ultimately inhibit cellular protein secretion. This motif constitutes a new target for the design of anti-viral drugs against noroviruses, as it is necessary for the antagonistic activity of p22 and is highly conserved in human noroviruses.

## Materials and Methods

### Vectors and cell lines

All genes were cloned using Gateway technology (Invitrogen) and expressed in pcDNA-DEST53 after sequence verification. Mutagenesis was performed using the QuikChange Multi Site-Directed Mutagenesis Kit (Stratagene). HEK-293T and Huh7 cells were maintained in DMEM supplemented with 10% fetal bovine serum. 293T cells were grown on plastic or coverslips coated with 10 µg/ml poly-D-lysine (Sigma). Transfection was carried out using Lipofectamine 2000 (Invitrogen) as per the manufacturer's instructions. cDNA encoding poliovirus 3A protein was obtained from Richard Lloyd of Baylor College of Medicine.

### Transfection of NV RNA

NV RNA was purified from volunteer stool samples and transformed into Huh7 cells as described previously [30]. Briefly, virus was purified from volunteer stool samples by sucrose cushion and cesium chloride gradient extraction, following which viral RNA was extracted using the QIAamp Viral RNA Mini Kit (Qiagen) following the manufacturer's instructions. Five hundred nanograms of purified viral RNA were then transfected into cells, which were then cultured for the indicated period of time.

### Immunofluorescence and antibodies

Cells were grown on cover slips and, following the indicated treatments, washed in PBS and subsequently fixed with 4% paraformaldehyde (Electron Microscopy Sciences, Hatfield, PA) in 0.1 M PBS and permeablized with 0.5% Triton X-100 in 0.1 M PBS. Cells were then washed in PBS and blocked with 1% bovine serum albumin (Sigma, St. Louis, MO) for 1 hour at 37°C, incubated overnight at 4°C with primary antibody in 0.1 M PBS, 1% BSA. Anti-human golgin-97 mouse monoclonal antibody was obtained from Molecular Probes (Invitrogen). GM130 mouse monoclonal antibody was obtained from BD Transduction Laboratories (San Jose, CA). Calnexin mouse monoclonal antibody and rabbit polyclonal antibody against β-COP were obtained from Affinity Bioreagents (Golden, CO). Rabbit polyclonal antibody against Sec24C was the generous gift of Bill Balch of Scripps Research Institute and has been previously described [59]. Rabbit polyclonal antibody against VP1 was also previously described [60]. Mouse monoclonal antibody 8B6 against placental alkaline phosphatase was obtained from AbCam (Cambridge, MA). Rabbit polyclonal antibody against protein disulfide isomerase (H-160) was obtained from Santa Cruz Biotechnology, Inc (Santa Cruz, CA). After incubation with primary antibody, cover slips were washed and incubated at room temperature for one hour with the corresponding AlexaFluor 488-, 594-, or 647-conjugated secondary antibodies (Invitrogen). Nuclei were stained with 300 nM DAPI (Invitrogen) at room temperature for 5 minutes. Cells were then washed and mounted using the ProLong Gold antifade reagent (Invitrogen). All images shown are representative of at least three independent experiments and the direct observation of no less than 50 cells with GFP expression levels in approximately the middle 70^th^ percentile.

### Fluorescence deconvolution microscopy

Deconvolution microscopy was performed with a Zeiss AxioVert S100 TV microscope and a DeltaVison restoration microscopy system (Applied Precision, Inc.) and imaged using either a 63X objective lens (1.40 NA). A *z* series of focal planes was digitally imaged and deconvolved with the Delta-Vision constrained iterative algorithm to generate high-resolution images, from which Quick Projections were obtained. Brightness and contrast levels were adjusted appropriately in Adobe Photoshop version CS2.

### Flow-coupled electron microscopy

293T cells were transfected with the indicated plasmids and at 24 hpt were harvested in 0.1 M PBS containing 1% BSA. Cells were then flow sorted for GFP on a BD SORP FACSAria II flow cytometric cell sorter with elution into 10% DMEM. Cells were then centrifuged at 500×g for 10 minutes, suspended in 10% DMEM and plated onto plastic dishes coated with 10 µg/ml poly-D lysine. Twenty-four hours later, and therefore 48 hpt, all cells were validated to be GFP positive under an epifluorescence microscope. Cells were then rinsed once in 0.1 M PBS and fixed in Karnovsky's Fixative (2% formaldehyde, 2.5% glutaraldehyde in 0.1 M cacodylate buffer +2 mM CaCl_2_, pH 7.4) for 1 hour on ice. After being held overnight at 4°C in weak fix (1 part Karnovsky's Fixative: 10 parts 0.1 M cacodylate buffer +2 mM CaCl_2_), the cells were rinsed 3 times in 0.1 M cacodylate buffer +2 mM CaCl_2_, then post-fixed in 1% OsO_4_ in 0.1 M cacodylate at 4°C. After 3 rinses in cacodylate buffer, cells were dehydrated in a gradient series of ethanols from 30–50%, en bloc stained with saturated uranyl acetate in 50% ethanol for 1 hour, carried through to 100% ethanol, then infiltrated in 1 part 100% ethanol: 1 part Spurr's Low Viscosity Resin overnight at room temperature. The rest of the infiltration was performed the next day, through 3 changes of pure resin. A small amount of pure resin was placed in the bottom of thoroughly drained plates, and the plated cells and resin were cured at 60°C for 3 days. Sections were cut at 70–80 nm using a Diatome diamond knife and an RMC MT6000-XL ultramicrotome. Sections were then collected on 100–150 mesh copper grids, counter-stained with Reynold's lead citrate and viewed on a Hitachi H-7500 transmission electron microscope. Image brightness and contrast were adjusted appropriately in Adobe Photoshop version CS2.

### Cytosolic and membrane fractionation

Transfected 293T cells were suspended in 0.5 ml of homogenization buffer [200 mM HEPES (pH 7.5), 5 mM sodium pyrophosphate, 5 mM EGTA, 1 mM MgCl_2_, 1 mM sodium orthovanadate, 50 µM leupeptin, 200 µM PMSF, 1 µM pepstatin A], followed by sonication and centrifugation at 100,000×g for one hour at 4°C, and the resulting supernatant was the cytosolic fraction. Pellets were resuspended in 0.5 ml extraction buffer [20 mM Tris-HCl (pH 7.5), 1% Triton X-100, 100 mM NaCl, 1 mM MgCl_2_, 1 mM CaCl_2_, 5 mM NaF, 1 mM sodium orthovanadate, 50 µM leupeptin, 200 µM PMSF, 1 µM pepstatin A], followed by incubation with rotation at 4°C for one hour and centrifuged at 100,000×g for 1 hour and the supernatant was collected as the membrane fraction. Fractions were then precipitated by addition of trichloroacetic acid to 10% and centrifugation at 16,000×g for 5 minutes. The pellet was then washed in acetone, pelleted, and resuspended in 50 µl of 5X SDS-PAGE sample buffer. Lysate aliquots were boiled for 3 minutes and analyzed by electrophoresis on 12.5% SDS-PAGE gels. Separated proteins were transferred from the gel to nitrocellulose membrane (Amersham Biosciences) and membranes were blocked in 5% Blotto (5% fat-free Carnation milk in 0.01 M PBS) and incubated with a mouse anti-GFP monoclonal antibody (Clontech, Mountain View, CA) in 0.5% Blotto overnight at RT. Primary antibody was removed and membranes were washed three times with 0.5% Blotto. Horseradish peroxidase-conjugated secondary goat anti-mouse immunoglobulin G antibody (Sigma-Aldrich) was incubated with the membranes for ∼2 h at RT and subsequently washed three times with 0.5% Blotto. Membranes were developed with SuperSignal sensitivity substrate (Pierce).

### SEAP assay

The plasmid pCMV-UTR-SEAP was obtained from J. Lindsay Whitton (Scripps Research Institute) [28] and was made into a Gateway-competent vector using the Gateway Vector Conversion System (Invitrogen) at the *NotI* site of the vector. 293T cells were grown to ∼60% confluence in 24 well plates and transfected. Two hours before the indicated time points, cells were washed 2 times in 0.01 M PBS and the media was replaced with 400 µl of fresh media. At the indicated time points, the media containing “extracellular SEAP” was removed. At this same time point, “intracellular SEAP” values were obtained by washing cells once in cold 0.01 M PBS and adding 400 µl of media containing 0.5% Triton X-100 to lyse cells and solubilize any SEAP that had been retained in cells, and then incubating cells at room temperature for ∼5 minutes. Lysed cells were then collected and spun at 13,000×g for one minute to remove cellular debris. Enzymatic SEAP activity in “intracellular” and “extracellular” fractions was assayed with the PhosphaLight TM System (Applied Biosystems), and overall Secreted SEAP was calculated with the equation: Secreted SEAP  =  (SEAP_extracellular_/(SEAP_extracellular_ + SEAP_intracellular_))×100 [28].

### EndoH Sensitivity Assay

Sensitivity of VSV G and chimeras to digestion with endoglycosidase H was carried out as previously described [9], [40], [42] with minor modifications. VSV G or chimeras were expressed in 293T cells from the plasmid pMD2.G. Twenty-four hours post-transfection, cells were washed 3X in Cys/Met-free DMEM and incubated at 37°C for 15 minutes. Cells were then labeled with 200 µCi/ml ^35^S-Met (Amersham BioSciences) for 15 minutes at 37°C, washed 3X in DMEM containing 10% FBS, and 400 µl of DMEM containing 10% FBS was added to cells, which were then incubated at 37°C. At the indicated time post-pulse, cells were washed 1X in cold PBS and harvested in VSV G Lysis Buffer (50 mM Tris pH 8, 62.5 mM EDTA, 1% NP-40, 0.4% deoxycholate, 50 µM leupeptin, 200 µM PMSF, 1 µM pepstatin A). Supernantants were cleared with 5 µl of Protein A Magnetic Beads (Invitrogen) and incubated overnight with monoclonal antibody against the luminal domain of VSV G (clone 8G5) [42], generously provided by Bill Balch, Scripps Research Institute. Immuno-complexes were collected by magnetic capture and washed 2X in VSV G Lysis Buffer, resuspended in 40 µl of 2.5X SDS-PAGE Sample Buffer and boiled for 5 minutes. Half of the IP was digested with 1 µl of endoglycosidase H (EndoH) (1 U/200 µl, Roche) at 37°C for 1 hour. EndoH treated and non-treated samples were then run on a 7.5% SDS-PAGE gel, transferred to nitrocellulose, and bands were quantitated following scanning on a Typhoon Trio Variable Mode Imager (GE Healthcare) and ImageQuant 5.1 to quantitate band intensity.

### Statistical analysis

Two-tailed, unpaired Student's *t*-tests assuming unequal variance were used to determine statistical significance; p values are indicated where appropriate.

## Supporting Information

Figure S1 NV p22 initially localizes to and induces disruption of the trans Golgi. Cells expressing GFP or GFP-tagged Norwalk virus (NV) p22 were immuno-stained for the trans Golgi marker protein golgin-97 (Alexa 594-conjugated secondary antibody, red fluorescence) at the indicated times post-transfection. Nuclei were stained with DAPI (blue fluorescence) and cells were imaged by deconvolution microscopy.
(2.88 MB TIF)

Figure S2 Equivalent amounts of protein are made during SEAP analysis of p22 constructs containing alanine mutations within the predicted ER export signal. Proteins in lysates from the 36 hpt intracellular fraction of cells utilized in the indicated SEAP assay were run on a 4–20% SDS-PAGE gel and detected with monoclonal antibody against either GFP or actin by western blot.
(0.74 MB TIF)

Figure S3 Calnexin is an appropriate marker of the endoplasmic reticulum in 293T cells. Non-transfected 293T cells were fixed and immuno-stained for the ER marker proteins calnexin and protein disulfide isomerase with mono- (red fluorescence, Alexa594-conjugated secondary antibody) and poly-clonal antibody (green fluorescence, Alexa488-conjugated secondary antibody), respectively. Nuclei were stained with DAPI (blue fluorescence) and cells were imaged by deconvolution microscopy.
(3.02 MB TIF)
