# Supplementary figures and images for: Inhibition of Cellular Protein Secretion by Norwalk Virus Nonstructural Protein p22 Requires a Mimic of an Endoplasmic Reticulum Export Signal
Source: PLoS One. 2010 Oct 18;5(10):e13130. doi: 10.1371/journal.pone.0013130 (PMC2956632; doi:10.1371/journal.pone.0013130)

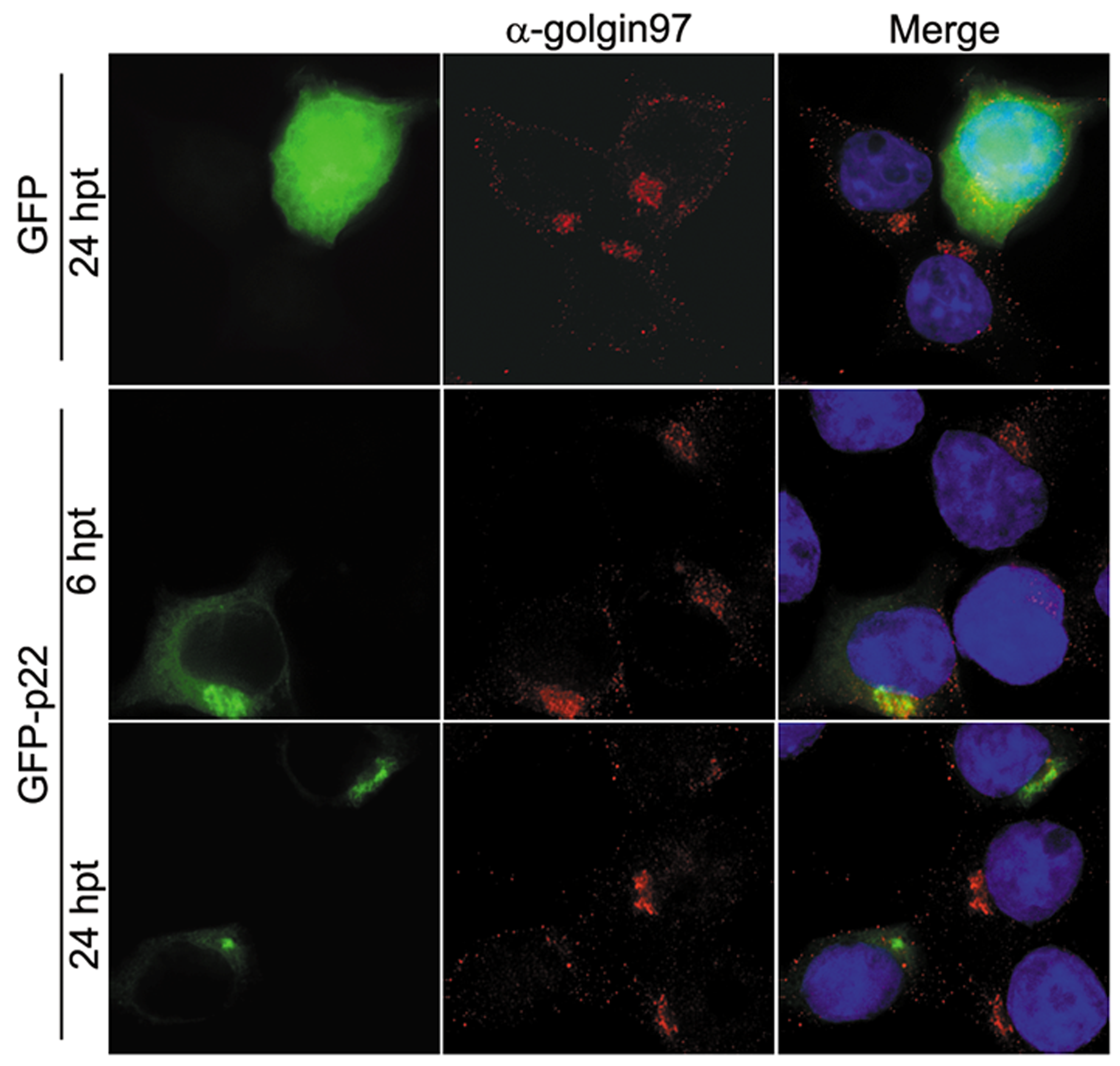

Supplement: Figure S1 — NV p22 initially localizes to and induces disruption of the trans Golgi. Cells expressing GFP or GFP-tagged Norwalk virus (NV) p22 were immuno-stained for the trans Golgi marker protein golgin-97 (Alexa 594-conjugated secondary antibody, red fluorescence) at the indicated times post-transfection. Nuclei were stained with DAPI (blue fluorescence) and cells were imaged by deconvolution microscopy. (2.88 MB TIF) [file pone.0013130.s001.tif]

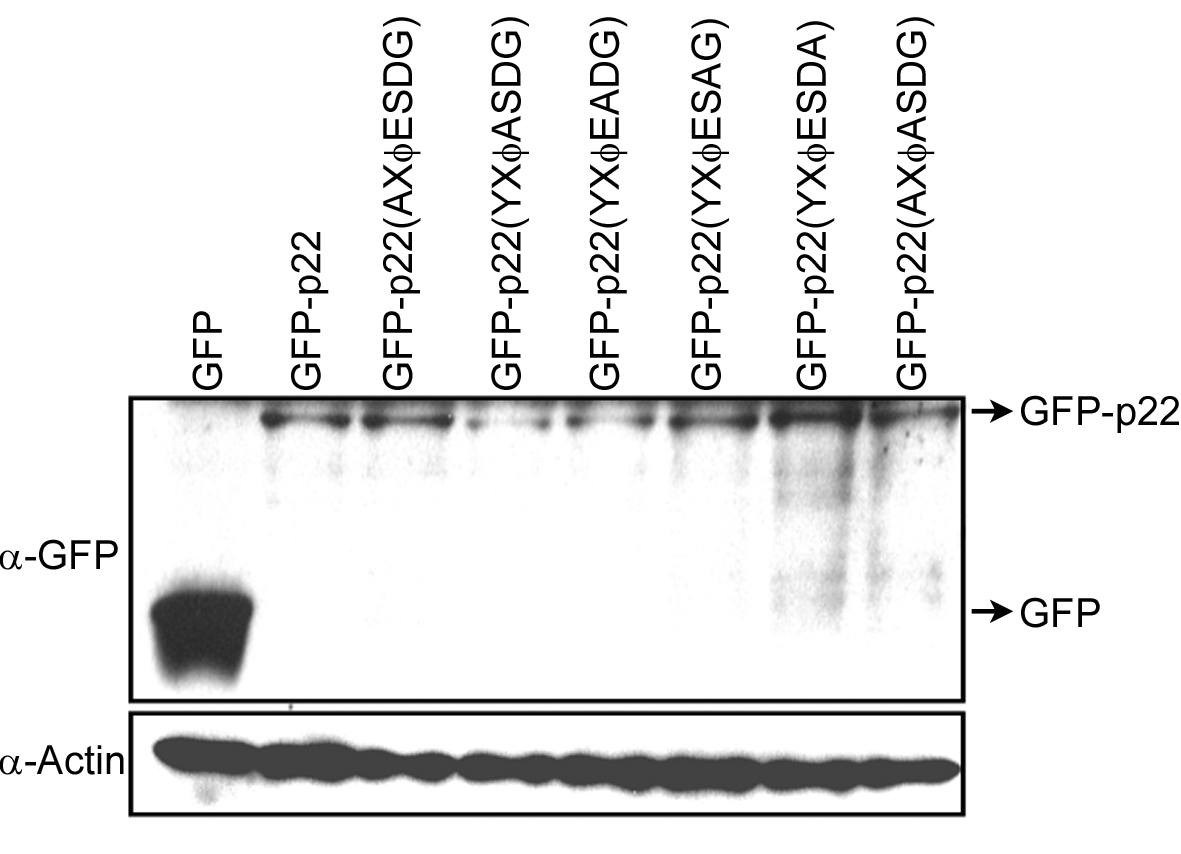

Supplement: Figure S2 — Equivalent amounts of protein are made during SEAP analysis of p22 constructs containing alanine mutations within the predicted ER export signal. Proteins in lysates from the 36 hpt intracellular fraction of cells utilized in the indicated SEAP assay were run on a 4–20% SDS-PAGE gel and detected with monoclonal antibody against either GFP or actin by western blot. (0.74 MB TIF) [file pone.0013130.s002.tif]

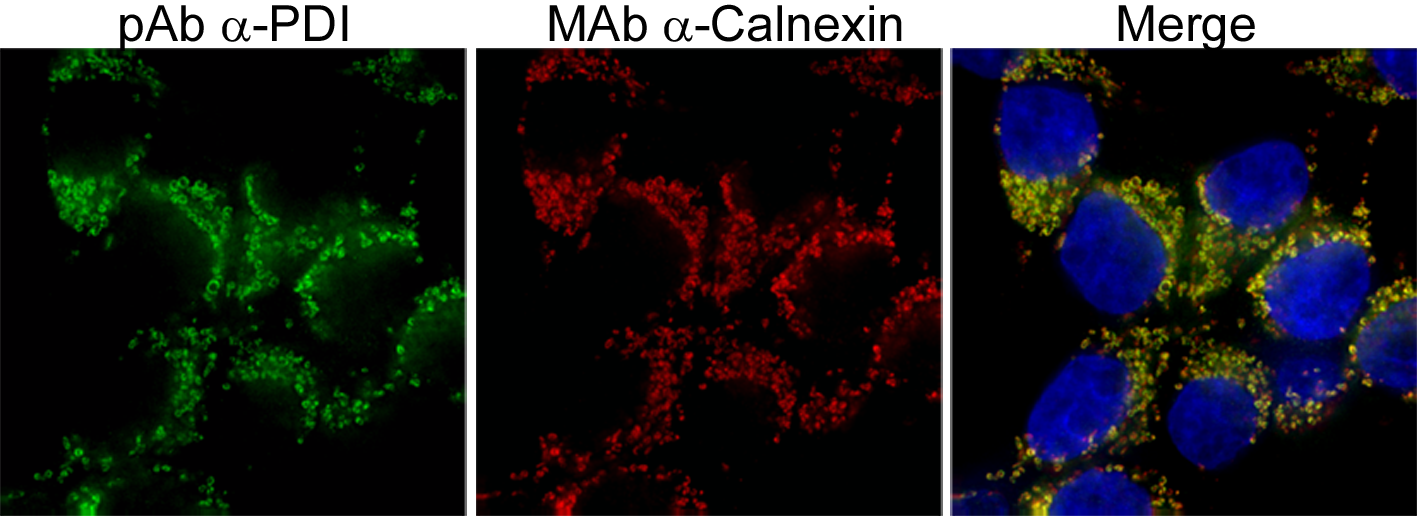

Supplement: Figure S3 — Calnexin is an appropriate marker of the endoplasmic reticulum in 293T cells. Non-transfected 293T cells were fixed and immuno-stained for the ER marker proteins calnexin and protein disulfide isomerase with mono- (red fluorescence, Alexa594-conjugated secondary antibody) and poly-clonal antibody (green fluorescence, Alexa488-conjugated secondary antibody), respectively. Nuclei were stained with DAPI (blue fluorescence) and cells were imaged by deconvolution microscopy. (3.02 MB TIF) [file pone.0013130.s003.tif]
